# A Highly Hydrophilic and Biodegradable Novel Poly(amide-imide) for Biomedical Applications

**DOI:** 10.3390/polym8120441

**Published:** 2016-12-19

**Authors:** Qiying Zou, Qian Zhou, Langlang Liu, Honglian Dai

**Affiliations:** State Key Laboratory of Advanced Technology for Materials Synthesis and Processing, Wuhan University of Technology, Wuhan 430070, China; zqy1123937213@whut.edu.cn (Q.Z.); effier@whut.edu.cn (Q.Z.); 243654@whut.edu.cn (L.L.)

**Keywords:** poly(amide-imide) (PAI), l-glycine, degradation, hydrophilicity, biocompatibility

## Abstract

A novel biodegradable poly(amide-imide) (PAI) with good hydrophilicity was synthesized by incorporation of l-glycine into the polymer chain. For comparison purposes, a pure PAI containing no l-glycine was also synthesized with a three-step method. In this study, we evaluated the novel PAI’s thermal stability, hydrophilicity, solubility, biodegradability and ability to support bone marrow mesenchymal stem cell (BMSC) adhesion and growth by comparing with the pure PAI. The hydrophilic tests demonstrated that the novel PAI has possible hydrophilicity at a 38° water contact angle on the molecule surface and is about two times more hydrophilic than the pure PAI. Due to an extra unit of l-glycine in the novel PAI, the average degradation rate was about 2.4 times greater than that of the pure PAI. The preliminary biocompatibility studies revealed that all the PAIs are cell compatible, but the pure PAI exhibited much lower cell adhesion than the l-glycine-incorporated novel PAI. The hydrophilic surface of the novel PAI was more suitable for cell adhesion, suggesting that the surface hydrophilicity plays an important role in enhancing cell adhesion and growth.

## 1. Introduction

The use of implants for restoring the structure and function of damaged bones is now the main therapy in orthopedic surgery [1]. As implant materials, one of the most widely used polyetheretherketones (PEEK) is biocompatible, chemically stable, radiolucent and has an elastic modulus similar to that of human bones [2,3,4]. However, PEEK is not biodegradable along with the absorption of bone graft at the later stage of implantation, which may result in stress shielding for bone grafts and slow down the fusion rate in PEEK clinical applications [5,6]. On the other hand, the PEEK polymer is biologically inert because of its hydrophobic surface, which may induce the deposition of a peri-implant fibrous capsule preventing bone deposition [7]. With other factors, the complication incidences caused by long-term existence of surgical implants have shown an increased trend recently. However, most synthetic biodegradable polymer implants such as poly(l-lactic acid) (PLLA) and poly(lactic-*co*-glycolic acid) (PLGA) have limited cell affinity, which is related to their hydrophobic surface [8]. Thus, biodegradable and hydrophilic implants seem more suitable for bone repair in orthopedic surgery [7,8,9,10].

The incorporation of l-amino acids into a polymeric molecular chain has attracted significant attention because of the broad applications of biodegradable polymers in biomedical, drug-delivery, pharmaceutical and tissue engineering [11,12,13,14]. Many studies have shown that non-biodegradable polymers become biodegradable when incorporated with l-amino acid [15,16,17,18,19,20]. The reason why l-amino acid-incorporated polymers are biodegradable is thought to be that the incorporation of l-amino acid may be targeted for cleavage by proteases [21,22,23]. Moreover, polymers such as poly(l-lactic acid-*co*-l-lysine) and poly(γ-methyl-l-glutamate) modified by incorporation of hydrophilic l-amino acid are expected to show improved hydrophilicity and cell adhesion [24,25].

Poly(amide-imide)s (PAIs) containing both flexible amide and thermal tolerable imide groups along the main chain of the polymer backbone are a kind of amorphous thermoplastic resin and have preeminent heat resistance, excellent mechanical properties, outstanding oxidation resistance and hydrogen bonding interaction, making them a promising matrix candidate for hybrid materials [26,27,28,29,30]. Of the polymer hybrid composites investigated, the l-amino acids incorporated poly(amide-imide) composites are of particular interest. The poly(amide-imide)s containing *N*-trimellitylimido-l-amino acid and 5-(2-benzimidazole)-1,3-phenylenediamine synthesized by S. Mallakpour and M. Khani et al. not only produce prominent performance of PAIs, but are also potentially biocompatible and biodegradable [31].

Based on the above factors, we incorporate the hydrophilic l-glycine unit into the polymer backbone, aiming to synthesize a hydrophilic and biodegradable novel PAI to solve PEEK non-degradable and surface hydrophobic issues when applied to bone repair implants. The other reason that l-glycine has been employed as the incorporated l-amino acid is due to its relatively fewer functional groups when compared to other hydrophilic l-amino acids, which would be expected to avoid the occurrence of unnecessary side reactions in the process of synthesis.

In this study, a hydrophilic l-glycine based novel PAI was designed and synthesized from *N*,*N*′-(4,4′-diphthaloyl)-*bis*-l-glycine via direct polycondensation with 4,4′-diaminodiphenyl ether (ODA) in the green medium of molten tetrabutylammonium bromide (TBAB) and triphenyl phosphite (TPP) under traditional heating. In addition, the non l-glycine containing pure PAI, with a structure similar to the novel PAI, was also synthesized by a three-step method for comparison purposes. We made comparisons of both the l-glycine incorporated novel PAI and the pure PAI from their thermal stability, hydrophilicity, solubility, biodegradability and biocompatibility, respectively. The in vitro biocompatibility of novel PAI and pure PAI was evaluated in terms of adhesion and proliferation of bone marrow mesenchymal stem cells (BMSCs).

## 2. Materials and Methods

### 2.1. Materials

l-glycine (CRS, 98.5%, *M*_w_ = 75.07 g·mol^−1^), 4,4′-diaminodiphenyl ether (ODA; AR, 98%, *M*_w_ = 200.24 g·mol^−1^), tetrabutylammonium bromide (TBAB; AR, 99%, *M*_w_ = 322.37 g·mol^−1^), triphenylphosphite (TPP; AR, 98%, *M*_w_ = 310.28 g·mol^−1^), 3,3′,4,4′-biphenyltetracarboxylic dianhydride (BPDA; AR, 97%, *M*_w_ = 294.22 g·mol^−1^), *N*,*N*-dimethylacetamide (DMAc; AR, 99%, d = 0.94 g·cm^−3^ at 20 °C, *M*_w_ = 87.12 g·mol^−1^) and 4,4′-Oxybis (benzoic acid) (AR, 98%, *M*_w_ = 258.23 g·mol^−1^) were purchased from Merck Chemical CO. (Seelze, Germany). FITC-phalloidin, Alkaline Phosphatase Assay Kit, 4′,6-diamidino-2-phenylindole (DAPI) and Cell Counting Kit-8 (CCK-8) were obtained from Sigma-Aldrich (St. Louis, MO, USA).

### 2.2. Techniques

Proton nuclear magnetic resonance (^1^H NMR, 400 MHz) spectra were recorded on a spectrometer (Bruker Avance 500, Bruker, Karlsruhe, Germany) at room temperature (RT) in *N*,*N*′-dimethylsulphoxide-*d*_6_ (DMSO-*d*_6_). Multiplicities of proton resonance were designated as singlet (s), doublet (d), triplet (t), and multiplet (m). FTIR spectra were recorded in the range of 4000–400 cm^−1^ on a Fourier transform infrared spectrometer (Nicolet 6700, Thermo Nicolet Corporation, New Castle, DE, America) in KBr. Band intensities were assigned as weak (w), medium (m), strong (s) and very strong (vs). Elemental analysis was made on a CHNS/O elemental analyzer (Vario EL Cube, Elementar, Munich, Germany). Molecular weight measurements were done on the gel permeation chromstography (GPC) instrument (Waters 1525 GPC, Shimadzu, Shimane, Japan) equipped with three Waters Styragel columns (HT3, HT4 and HT5) and a differential refractometer detector (Waters 2414, Shimadzu, Shimane, Japan). Dimethylformamide (DMF) was used as an eluent at 35 °C at a flow rate of 1 mL·min^−1^. Polystyrene was used as the standard. Thermal gravimetric analysis (TGA) was performed by a simultaneous thermal analyzer (STA 503, Baehr, Ochtrup, Germany) at a heating rate of 10 °C·min^−1^ from 25 to 800 °C under pure nitrogen atmosphere.

### 2.3. Synthesis of the l-Glycine Based Novel PAI

#### 2.3.1. Synthesis of *N*,*N*′-(4,4′-Diphthaloyl)-*bis*-l-glycine (Scheme 1)

l-glycine 0.3003 g (4 mmol) and BPDA 0.5884 g (2 mmol) were added to a 50 mL flask containing 15 mL of acetic acid under stirring. The mixture was stirred at 25 °C overnight and then refluxed for 5 h at 118 °C. After that, the solvent was removed under reduced pressure, and the residue was washed with 30 mL of 5% hydrochloric acid aqueous solution to form the white precipitate, filtered and dried at 60 °C in vacuum for 12 h to yield *N*,*N*′-(4,4′-diphthaloyl)-*bis*-l-glycine.

The following are the characterizations:

FTIR (KBr): 3081 (m, carboxyl *v*_OH_), 2943 (m, *v*_as_ –CH_2_–), 1778 (s, imide *v*_as_ C=O), 1720 (vs, imide *v*_s_ C=O), 1429, 1405, 1384 (s, imide *v*_C–N_), 1231 (s, carboxyl *v*_co_), 1122 (m, *v*_Ar–O–Ar_), 753 (s, γ_Ar–H_) cm^−1^ (Figure S1).

^1^H NMR (400 MHz, DMSO-*d*_6_, δ, ppm): 4.362 (s, 2H, –CH_2_–), 8.051–8.067 (d, 5H, Ar–H), 8.331–8.347 (d, 4H, Ar–H), 8.380 (s, 3H, Ar–H), 13.259 (s, 1H, –COOH) ppm (Figure S2).

Elemental analysis: Found: C, 57.21; H, 2.96; N, 6.89%; C_20_H_12_N_2_O_8_ requires C, 58.82; H, 2.94; N, 6.86%.

#### 2.3.2. Synthesis of the Novel PAI

*N*,*N*′-(4,4′-diphthaloyl)-*bis*-l-glycine 0.864 g (2 mmol) and 4,4′-diaminodiphenyl ether 0.4005 g (2 mmol) were added to a flask containing molten TBAB. Then 4.9645 g of TPP was added drop-wise to the mixture which was stirred until a homogeneous solution was formed. After that, the solution was stirred at 110 °C for 2.5 h in a pure nitrogen atmosphere and then the viscous solution was precipitated in 300 mL of anhydrous ethanol, filtered and dried at 60 °C in vacuum for 12 h to yield yellow powders of novel PAI.

### 2.4. Synthesis of the Pure PAI

#### 2.4.1. Preparation of 4,4′-Oxidation *bis* Ethly Benzoate (See Step 1 in Scheme 2)

5.1646 g of 4,4′-Oxybis (benzoic acid) (0.02 mol) and 9.2140 g of anhydrous ethanol (0.1 mol) were placed in a 100 mL flask. Then 14.6 mL of SOCl_2_ was added drop-wise under stirring at 0~10 °C for 2 h. After the complete addition, the reaction mixture was heated up to 78 °C and refluxed for 3 h. Then, the incomplete reaction anhydrous ethanol and SOCl_2_ were distilled under atmospheric pressure to obtain crude ester. The crude ester was washed twice with 100 mL of 10% NaCO_3_ and 100 mL of deionized water, then was cooled in crushed ice to form a milky white needle crystal, dried at 25 °C for 12 h in vacuum to yield 4,4′-oxidation *bis* ethly benzoate.

The following are the characterizations:

FTIR (KBr): 3411 (w, float band *v*_C=O_), 3079 (w, *v*_Ar–H_), 2974 (m, *v*_as_ –CH_3_), 2933 (w, *v*_as_ –CH_2_–), 1716 (vs, *v*_C=O_), 1597, 1503 (s, *v*_–C=C–_), 1286, 1247 (vs, *v*_C–O–C_), 1164, 1102 (s, *v*_Ar–O–Ar_), 870, 852, 769 (m, γ_Ar–H_) cm^−1^ (Figure S1).

^1^H NMR (400 MHz, DMSO-*d*_6_, δ, ppm): 1.299–1.328 (t, 1H, –CH_3_), 4.286–4.328 (m, 2H, –CH_2_–), 7.172–7.189 (d, 3H, Ar–H), 8.001–8.017 (d, 4H, Ar–H) ppm (Figure S2).

Elemental analysis: Found: C, 68.92; N, 5.80%. Calcd. for C_18_H_18_O_5_: C, 68.79; O, 5.73%.

#### 2.4.2. Synthesis of 4,4′-Oxidation *bis* Benzoyl Hydrazine (See Step 2 in Scheme 2)

Synthetic 4,4′-oxidation *bis* ethly benzoate 6.28 g (0.02 mol), anhydrous ethanol 4.734 g (0.1 mol) and 85% hydrazine monohydrate 2.4028 g (0.048 mol) were put in a 100 mL flask. The reaction mixture was heated for 4 h at 80 °C under stirring, and was then cooled in the ice water bath to precipitate a white crystal. The white powder was dried at 25 °C for 12 h in vacuum to yield white solid 4,4′-oxidation *bis* benzoyl hydrazine.

The following are the characterizations:

FTIR (KBr): 3311 (s, *v* –NH_2_), 3078 (w, *v*_Ar–H_), 1708 (s, *v*_C=O_), 1622 (s, δ_N–H_), 1599, 1500 (s, *v*_–C=C–_), 1289, 1257 (s, *v*_C–N_), 1161, 1099 (m, *v*_Ar–O–Ar_), 868, 845, 764 (m, γ_Ar–H_) cm^−1^ (Figure S1).

^1^H NMR (400 MHz, DMSO-*d*_6_, δ, ppm): 4.294–4.315 (m, 1H, –NH_2_), 7.079–7.189 (m, 4H, Ar–H), 7.870–8.019 (m, 3H, Ar–H), 9.737 (s, 2H, –NH–) ppm (Figure S2).

Elemental analysis: Found: C, 59.51; H, 5.36; N, 19.21%. Calcd. for C_14_H_14_O_3_: C, 58.74; H, 4.89; N, 19.58%.

#### 2.4.3. Synthesis of the Pure PAI (See Step 3 in Scheme 2)

5.72 g of 4,4′-oxidation *bis* benzoyl hydrazine (0.02 mol) were dissolved in 60 mL of DMAc to form a homogeneous solution. Then 5.8844 g of BPDA (0.02 mol) was added to the solution by one-time and the reaction mixture was stirred for 12 h at 25 °C in a pure nitrogen atmosphere. After that, the reaction mixture was poured into 6 mL of acetic anhydride and 2.8 mL of triethylamine and was heated for 7 h at 80 °C. Then the mixture was precipitated in 300 mL of anhydrous ethanol, filtered and dried at 60 °C for 12 h in vacuum to yield yellow powders of pure PAI.

### 2.5. Hydrophilicity

Surface hydrophilicity of the novel PAI and the pure PAI were assessed by contact angle measurement using a contact angle testing device (JC 2000C 50 Hz, Dataphysics Co, Stuttgart, Germany). Deionized water was dropped on the surface of smooth and flat films, and three points at a distant of 5 mm were tested for each group of the novel PAI and the pure PAI.

### 2.6. In Vitro Degradation Assay

The degree of degradation was estimated from the weight loss of novel PAI and pure PAI samples over time in PBS buffer solution (pH 7.4). Furthermore, SEM (Hitachi S4800, Tokyo, Japan) was employed to analyze the surface morphology of PAI polymers during the degradation process. Each sample (diameter × height, 12 mm × 12 mm) of novel PAI and pure PAI was placed in sealed test tubes filled with PBS buffer solution. The weight to volume ratio of the samples to PBS was 1:20 [32]. Those tubes were placed in a 37 °C water bath with gentle shaking at approximately 70–80 rpm to simulate a dynamic in vivo environment [33]. At 1, 3, 5, 7, 14, 21 and 28 days from the initial incubation time, there specimens of each polymer were taken out and then dried at room temperature in vacuum for 3 days before analysis of weight loss. The degradation solution in each tube was collected for pH value measurement. At the same time, the PBS of the rest incubation samples was changed.

### 2.7. In Vitro Biocompatibility Assay

The in vitro biocompatibility of novel PAI and pure PAI was evaluated in terms of cell adhesion and proliferation of bone mesenchymal stem cells (BMSCs). Before the experiments, the specimens were sterilized by ethylene oxide for 2 h. The BMSCs were seeded onto the specimens with densities of 5 × 10^4^ cell/mL and 2 × 10^4^ cell/mL for cell adhesion and proliferation, respectively [34,35]. The cells were cultured in a medium consisting of an α-MEM (Gibco) supplemented with 10% fetal bovine serum (FBS) and 1% penicillin–streptomycin in a humidified incubator with 5% CO_2_ at 37 °C.

The morphology of the adhesive cells on novel PAI and pure PAI were examined using Confocal laser scanning microscope (CLSM) (IX71, Olympus Corporation, Tokyo, Japan). After 7 days of incubating, the each cell culture was douched with 100 μL PBS and then fixed by 100 μL 4% paraformaldehyde for 30 min. Then, the nucleus and cytomembrane were dyed at 20 min and 2 h with 200 μL 4′,6-diamidino-2-phenylindole (DAPI) and 200 μL FITC-phalloidin, respectively. Then the substrates were examined by CLSM.

The cell proliferation rate was examined using the CCK-8 method with the ISO standard. The control group contained no material extracts, while the experimental groups added two different extracts of materials. At 1, 3, 5, 7 days, the formazan product was quantified according to the light absorbance at the wavelength of 450 nm by using a microplate reader (Multiskan FC, Boston, MA, USA). The quantity of the formazan product was directly proportional to the number of living cells in the culture.

## 3. Results and Discussion

### 3.1. Polymer Synthesis

**Scheme 1:**
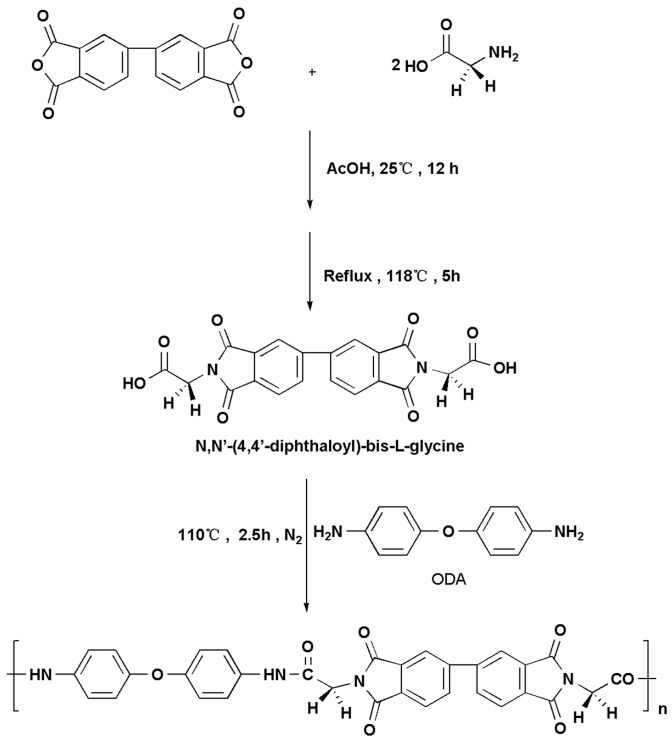
The synthetic scheme for the preparation of the novel PAI.

**Scheme 2:**
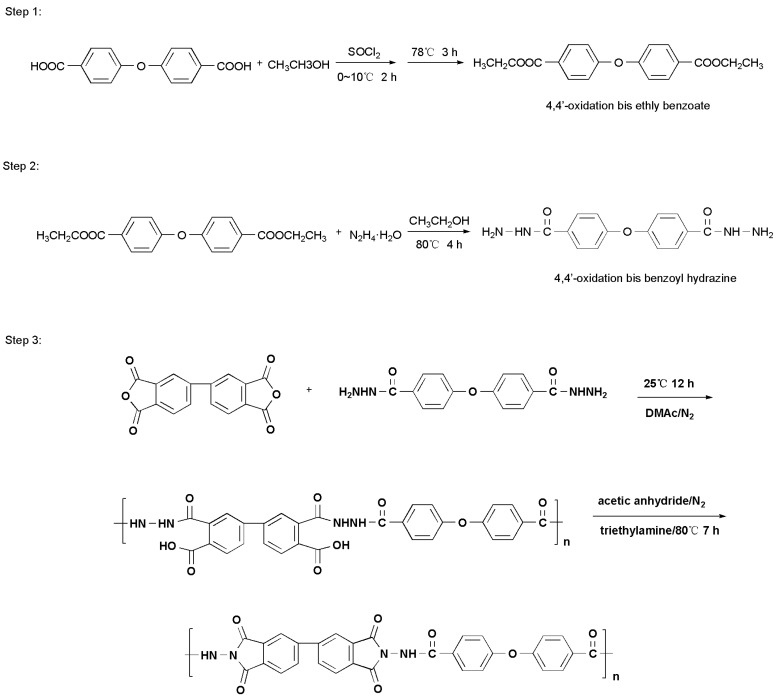
Schematic synthetic path for the pure PAI.

The schematic syntheses of the novel PAI and the pure PAI are shown in Scheme 1 and Scheme 2, respectively. The elemental analyses of the resulting novel PAI and pure PAI were inconsistent with the calculated values for the proposed structure (Table 1).

**Figure 1:**
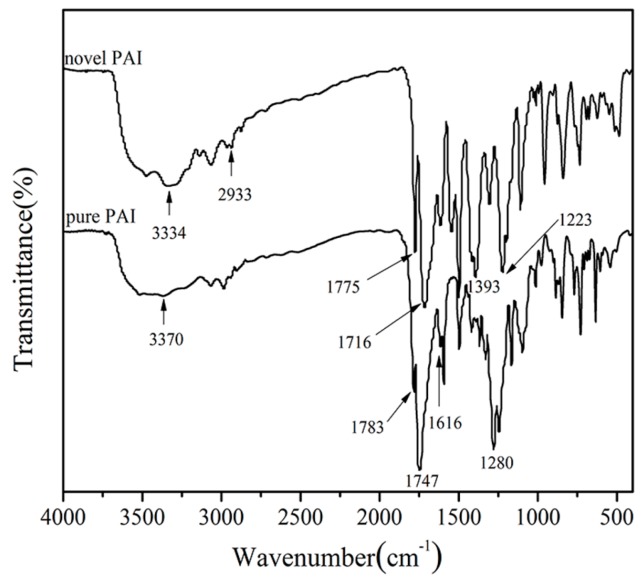
FTIR spectra of novel PAI and pure PAI.

Figure 1 displays the FTIR spectra of novel PAI and pure PAI. In the spectrum of novel PAI, absorption bands at 1775 and 1716 cm^−1^ (imide C=O asymmetrical and symmetrical stretching vibration), 2933 cm^−1^ (–CH_2_– asymmetrical stretching vibration), 1393 cm^−1^ (imide C–N stretching vibration), 1617 cm^−1^ (C=O stretching vibration for amide group, amide I band), 1546 and 3334 cm^−1^ (N–H bending vibration and stretching vibration for amide group, amide II band), 1223 cm^−1^ (amide C–N stretching vibration, amide III band), confirming the presence of imide rings and amide groups in the structure. The pure PAI showed its characteristic absorption bands at 1783 and 1747 cm^−1^ (imide C=O asymmetrical and symmetrical stretching vibration), 1616 cm^−1^ (C=O stretching vibration for amide group, amide I band), 3370 cm^−1^ (N–H stretching vibration for amide group, amide II band), 1280 cm^−1^ (amide C–N stretching vibration, amide III band).

**Figure 2:**
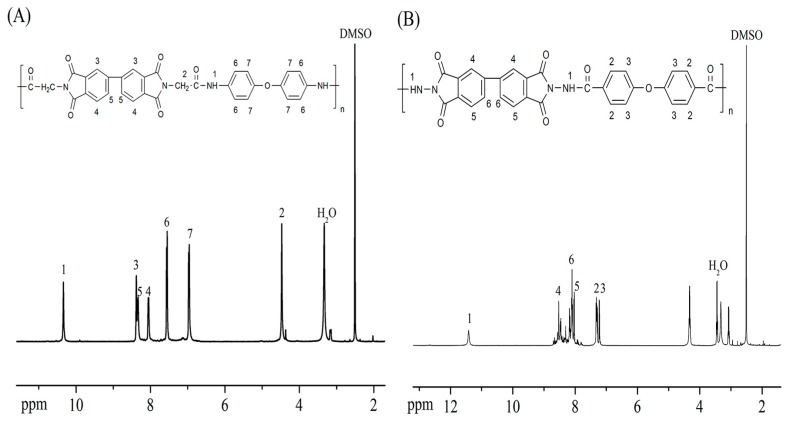
^1^H NMR spectra of novel PAI (**A**) and pure PAI (**B**).

In the ^1^H NMR spectrum of novel PAI, appearances of the N–H protons of amide group at 10.330 ppm demonstrated the existence of an amide group in the polymer backbone. The resonance of aromatic protons appeared in the range of 6.950–8.371 ppm and the peak at 4.465 ppm was attributable to the –CH_2_– (2) protons (Figure 2A). The pure PAI showed a peak at 11.411 ppm, which is attributable to the amide protons, and peaks at 7.207–8.518 ppm, which are attributable to the aromatic protons (Figure 2B). Above results confirmed that the novel PAI and pure PAI synthesized herein are in agreement with the purposed structure.

### 3.2. Solubility Property

The solubility results for novel PAI and pure PAI are summarized in Table 2. All the polymer samples were soluble in strong polar solvents such as NMP, DMAc, DMF and DMSO at room temperature or upon heating, and were insoluble in solvents such as CHCl_3_, methylbenzene and acetone. However, the pure PAI exhibited excellent solubility in less polar solvents of THF and pyridine just do not like the novel PAI, which can be explained by the fact that the incorporation of hydrophilic l-glycine had a certain influence on the solubility of the synthetic polymer.

### 3.3. Molecular Weight and Thermal Stability

The number-average molecular weights (*M*_n_) of the resulting polymers, novel PAI and pure PAI, were 26.062 and 24.631 kDa, with a polydispersity index (PDI) of 1.401 and 1.463, respectively, as determined by GPC using DMF as the eluent at 35 °C (shown in Table 3). Compared with the pure PAI, the novel PAI synthesized in the green medium of molten TBAB and TPP exhibited a narrower molecular weight distribution width, indicating the existence of higher purity structures in its polymer backbone.

**Figure 3:**
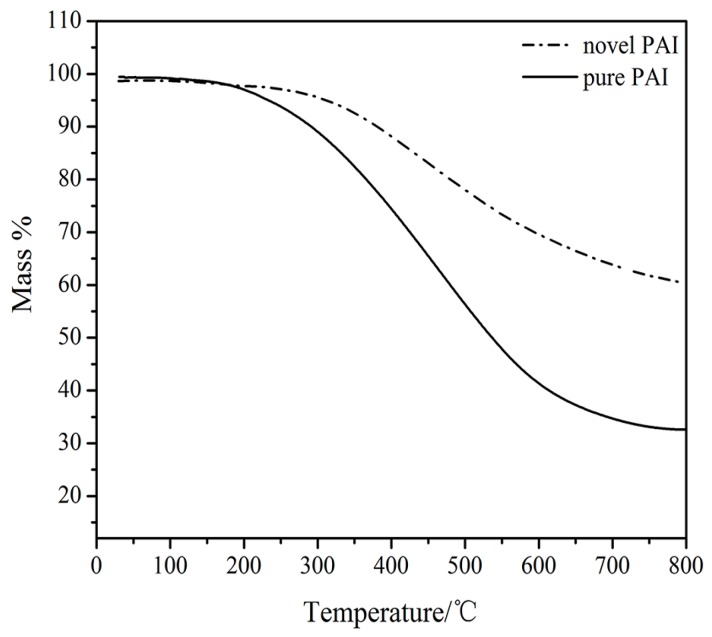
TGA thermograms of novel PAI and pure PAI.

As seen in Figure 3, the novel PAI showed more dramatic thermal stability than that of the pure PAI, suggesting that the l-glycine incorporation improved the thermal stability of the synthetic polymer. In general, the novel PAI with methylene connecting an amide group and an imide group in the polymer backbone was more difficult for thermal decomposition when compared to the pure PAI. The temperature of 5% and 10% weight loss for novel PAI and pure PAI are shown in Table 4.

### 3.4. Hydrophilicity Performance

**Figure 4:**
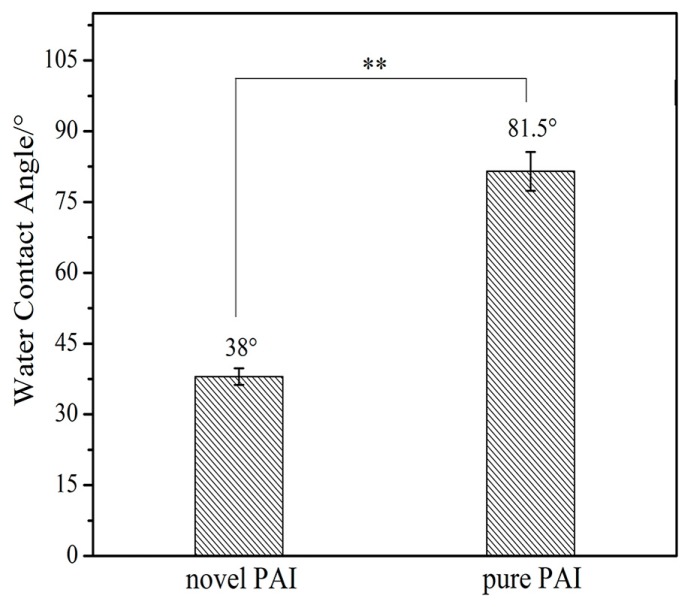
The water contact angle of the novel PAI and the pure PAI (** *p* < 0.01).

The water contact angle of the polymer films decreased from 81.5° to 38° after the l-glycine incorporation, suggestive of more hydrophilic surfaces (Figure 4). The lower water contact angle on the molecule surface of novel PAI may be related to hydrophilic l-glycine in its structure, which is consistent with the expected results when we designed the polymer structure. Moreover, the novel PAI showed lower contact angle compared to that of the PEEK (84.8°) and the biodegradable implants such as PLGA (74.5°) and PLLA (85°), manifesting the significantly improvement in surface hydrophilicity [7,8].

### 3.5. In Vitro Degradation Assay

**Figure 5:**
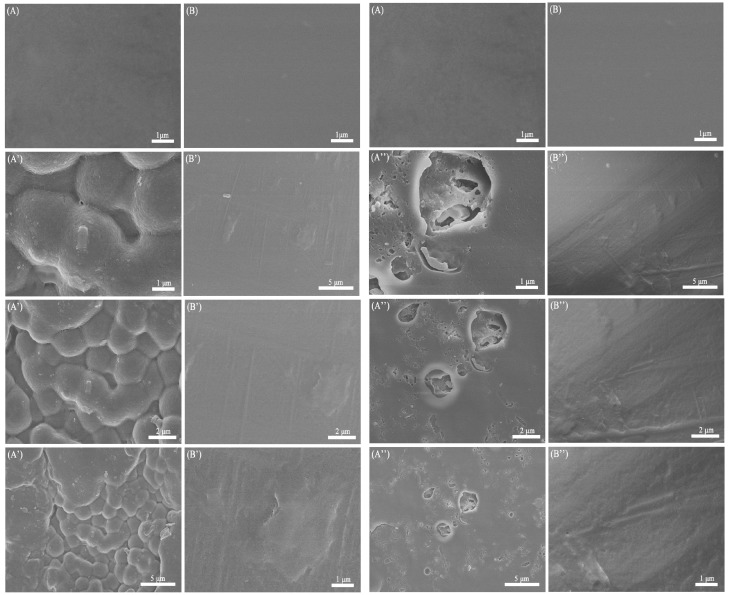
SEM images of novel PAI and pure PAI after 1, 3 days degradation at 37 °C in PBS. Before degradation ((**A**) novel PAI; (**B**) pure PAI); After degradation for 1 day ((**A**′) novel PAI; (**B**′) pure PAI); After degradation for 3 days ((**A**″) novel PAI; (**B**″) pure PAI).

**Figure 6:**
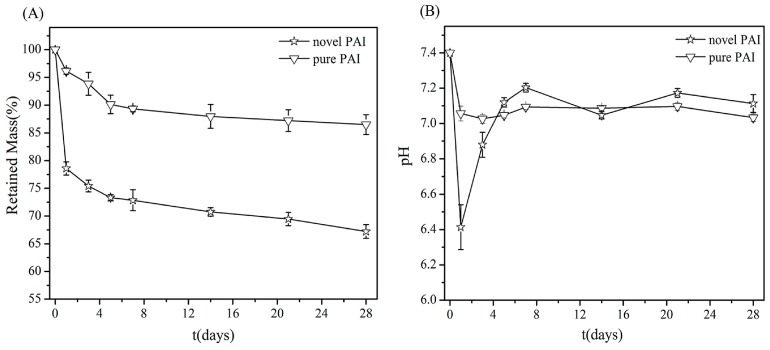
(**A**) Retained mass and (**B**) pH values of the degradation solution of novel PAI and pure PAI during the degradation process.

The surface morphology changes of novel PAI and pure PAI films upon degradation are shown in Figure 5. After incubating for 1 and 3 days, novel PAI showed a significant degradation as evident by the appearance of more surface erosion when compared to the pure PAI. The degradation data showed that the novel PAI degraded quickly and had a 21% weight loss on the first day and 33% weight loss after 28 days, about 2.4 times the average degradation rate of the pure PAI (Figure 6A). The data above support the view that the incorporation of l-glycine into the polymer chain can indeed improve the degradability of the synthetic polymer. In addition, the pH values of all degradation solution decreased in a certain extent, which may due to the degradation rate and the acidic substances produced during the degradation process (Figure 6B).

### 3.6. The In Vitro Biocompatibility Assay

**Figure 7:**
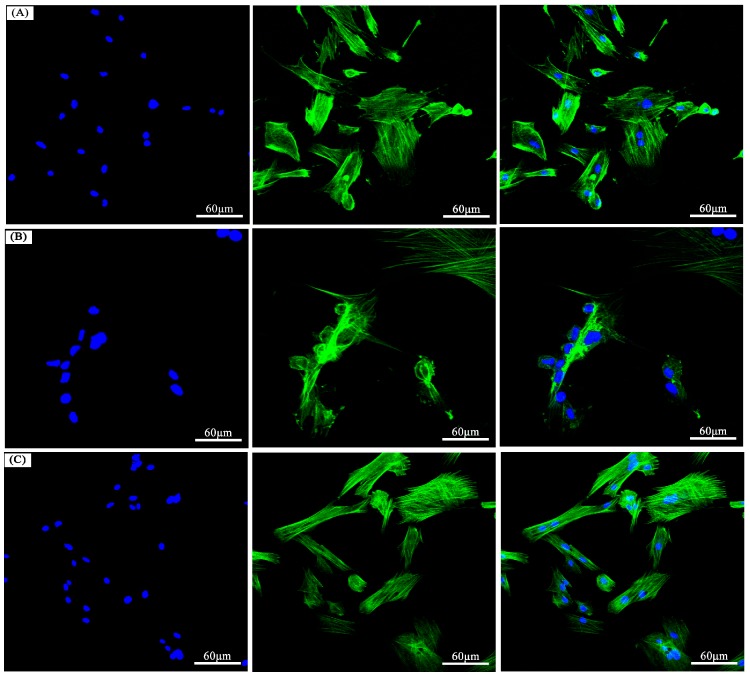
CLSM micrographs of the BMSCs that were cultured for 7 days on novel PAI and pure PAI ((**A**) novel PAI; (**B**) pure PAI; (**C**) empty control).

In the Figure 7, BMSCs were seeded and cultured for 7 days on the surfaces of novel PAI and pure PAI thin films, respectively. It can been seen that cells showed filopodia extensions and were well adhere to the novel PAI thin film. But for the pure PAI, cells exhibited round shapes and shrinkage morphology rather than extensions that not adhered to and spread on its surface were observed. Comparatively speaking, the hydrophilic surface of the novel PAI was more beneficial to initial cell adhesion when compared to that of the pure PAI.

**Figure 8:**
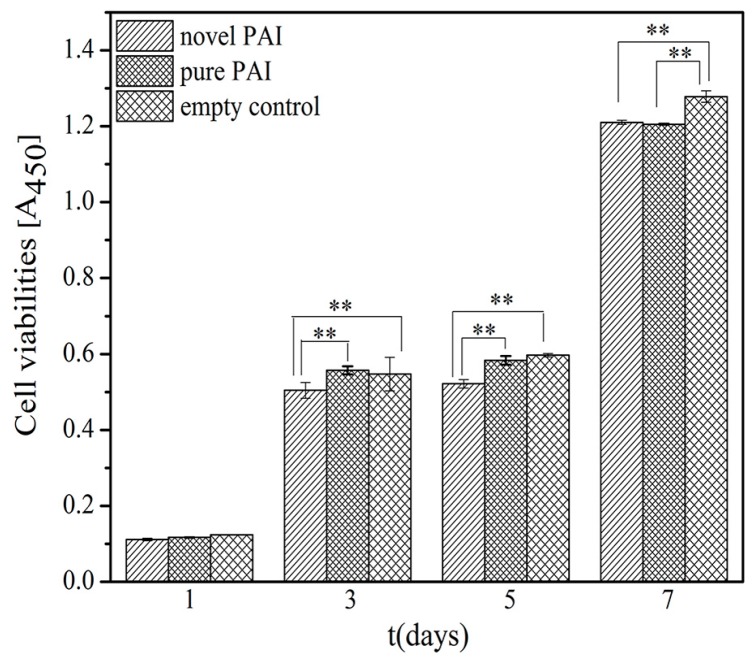
Cell viabilities of the BMSCs that were cultured for 1, 3, 5, 7 days on the novel PAI and the pure PAI, respectively (** *p* < 0.01).

**Figure 9:**
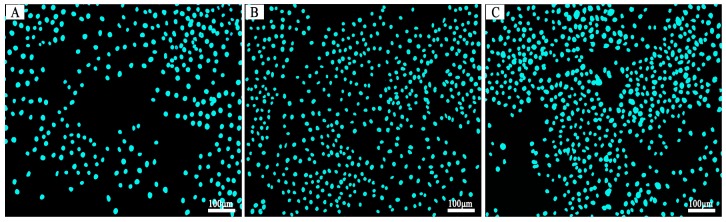
The proliferation conditions of BMSCs that were cultured for 3 days on novel PAI and pure PAI ((**A**) novel PAI; (**B**) pure PAI; (**C**) empty control).

The differences in cell proliferation on different materials after 1, 3, 5, 7 days cultivation were observed, as shown in Figure 8. BMSCs were proliferating on both novel PAI and pure PAI, indicating that they were cell compatible. Part of the nucleus were dyed with DAPI after 3 days of cultivation, as shown in the Figure 9. The number of cells that fluorescence micrographs showed were in accordance with the results measured by a microplate reader.

The in vitro biocompatibility assay findings suggested that the improvement in polymer hydrophilicity is conducive to adhesion and growth of BMSCs. However, further detailed assays of in vivo biocompatibility should also be carried out to evaluate the potential application of the polymers produced in this study as bone repair implants.

## 4. Conclusions

Our purpose was to synthesize a cell compatible polymer with acceptable hydrophilicity and degradability. We have successfully synthesized a novel biodegradable PAI that showed good hydrophilicity by introducing an l-glycine into the polymer chain. The pure PAI containing no l-glycine was also synthesized for comparison purposes. The novel PAI exhibited a higher hydrophilicity and degradability than those of the pure PAI. We also examined the in vitro biocompatibility of both the novel PAI and the pure PAI through some routine assays, and found that the hydrophilic novel PAI show some suitable characterizations for cell adhesion and growth compared to those of the pure PAI. The suitable properties of this novel PAI could potentially be used in orthopedic implants for biomedical applications.

## Figures and Tables

**Table 1:** Elemental analyses of novel PAI and pure PAI.

Polymer	Formula		C (%)	H (%)	N (%)
novel PAI	C_32_H_20_N_4_O_7_ (572.52)_n_	Calcd.	67.13	3.50	9.79
		Found	66.89	3.84	9.19
pure PAI	C_30_H_16_N_4_O_7_ (544.47)_n_	Calcd.	66.18	2.94	10.29
		Found	65.60	2.91	9.81

**Table 2:** Solubility of novel PAI and pure PAI ^a,b^.

Solvent	Novel PAI	Pure PAI
NMP	++	++
DMAc	++	++
DMF	++	++
DMSO	+	++
Pyridine	−	++
THF	−	++
CHCl_3_	−	−
CH_2_Cl_2_	−	−
Acetone	−	−
Methylbenzene	−	−
Dimethylbenzene	−	−

^a^ Solubility: ++, soluble at room temperature; +, soluble on heating; −, insoluble on heating; ^b^ Abbreviations: NMP, *N*-methyl-2-pyrrolidinone; DMAc, *N*,*N*-dimethylacetamide; DMF, *N*,*N*-dimethylformamide; THF, tetrahydrofuran; DMSO, dimethyl sulfoxide; CHCl_3_, chloroform; CH_2_Cl_2_, dichloromethane.

**Table 3:** Molecular weights of novel PAI and pure PAI (Figure S3).

Polymer	*M*_w_^a^ (kDa)	*M*_n_^a^ (kDa)	*M*_z_^a^ (kDa)	PDI	*M*_z_/*M*_w_
novel PAI	36.518	26.062	44.674	1.401	1.223
pure PAI	36.045	24.631	44.622	1.463	1.238

^a^ *M*_w_, *M*_n_, *M*_z_, PDI and *M*_z_/*M*_w_ of the polymers were estimated by GPC using polystyrene as standard in DMF.

**Table 4:** Thermal behavior of novel PAI and pure PAI.

Polymer	*T*_5_ (°C) ^b^	*T*_10_ (°C) ^b^
novel PAI	312	380
pure PAI	233	291

^b^ Temperature of 5% or 10% weight loss at a rating of 10 °C/min in a pure nitrogen atmosphere.

## Supplementary Materials

The supplementary materials are available online at www.mdpi.com/2073-4360/8/12/441/s1.
