# Supplementary material for: A Highly Hydrophilic and Biodegradable Novel Poly(amide-imide) for Biomedical Applications
Source: Polymers (Basel). 2016 Dec 19;8(12):441. doi: 10.3390/polym8120441 (PMC6432413; doi:10.3390/polym8120441)
Supplement: Supplementary file 1 [file polymers-08-00441-s001.pdf]

Supplementary Materials: A Highly Hydrophilic and Biodegradable Novel Poly(amide-imide) for Biomedical Applications

Qiying Zou, Qian Zhou, Langlang Liu and Honglian Dai

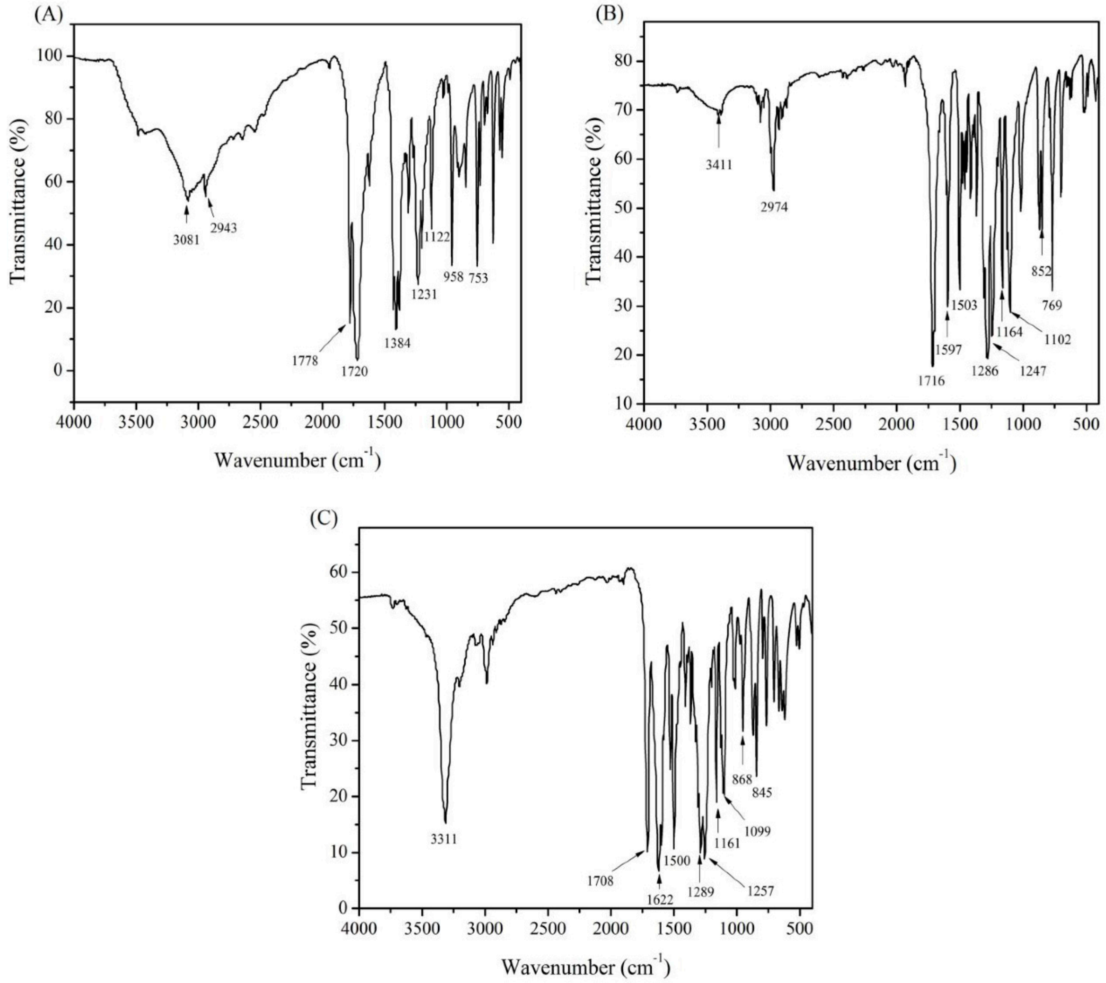

Figure S1. FT-IR spectra of (**A**) *N*,*N*′-(4,4′-diphthaloyl)-*bis*-L-glycine; (**B**) 4,4′-oxidation *bis* ethly benzoate and (**C**) 4,4′-oxidation *bis* benzoyl hydrazine.

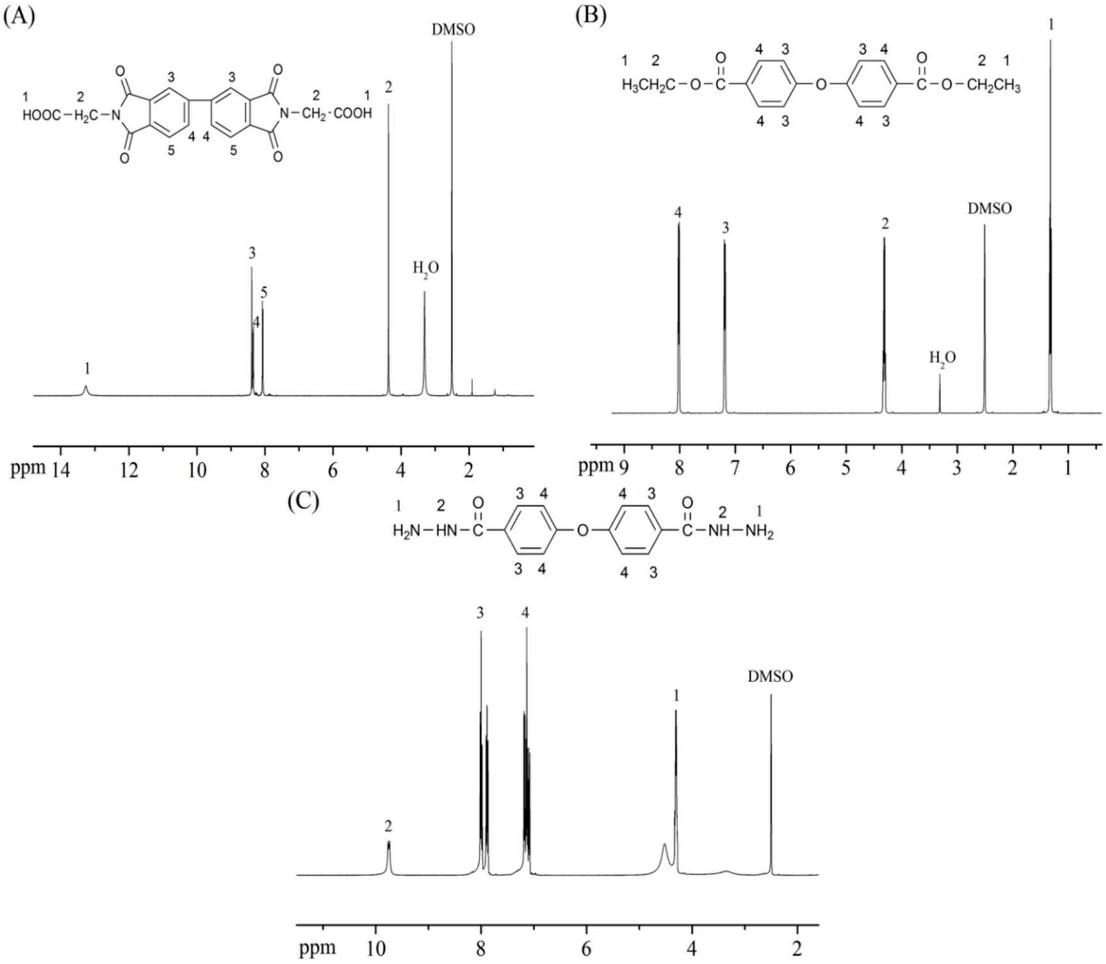

Figure S2. ^{1}H NMR spectra of (**A**) *N*,*N*′-(4,4′-diphthaloyl)-*bis*-L-glycine; (**B**) 4,4′-oxidation *bis* ethly benzoate and (**C**) 4,4′-oxidation *bis* benzoyl hydrazine.

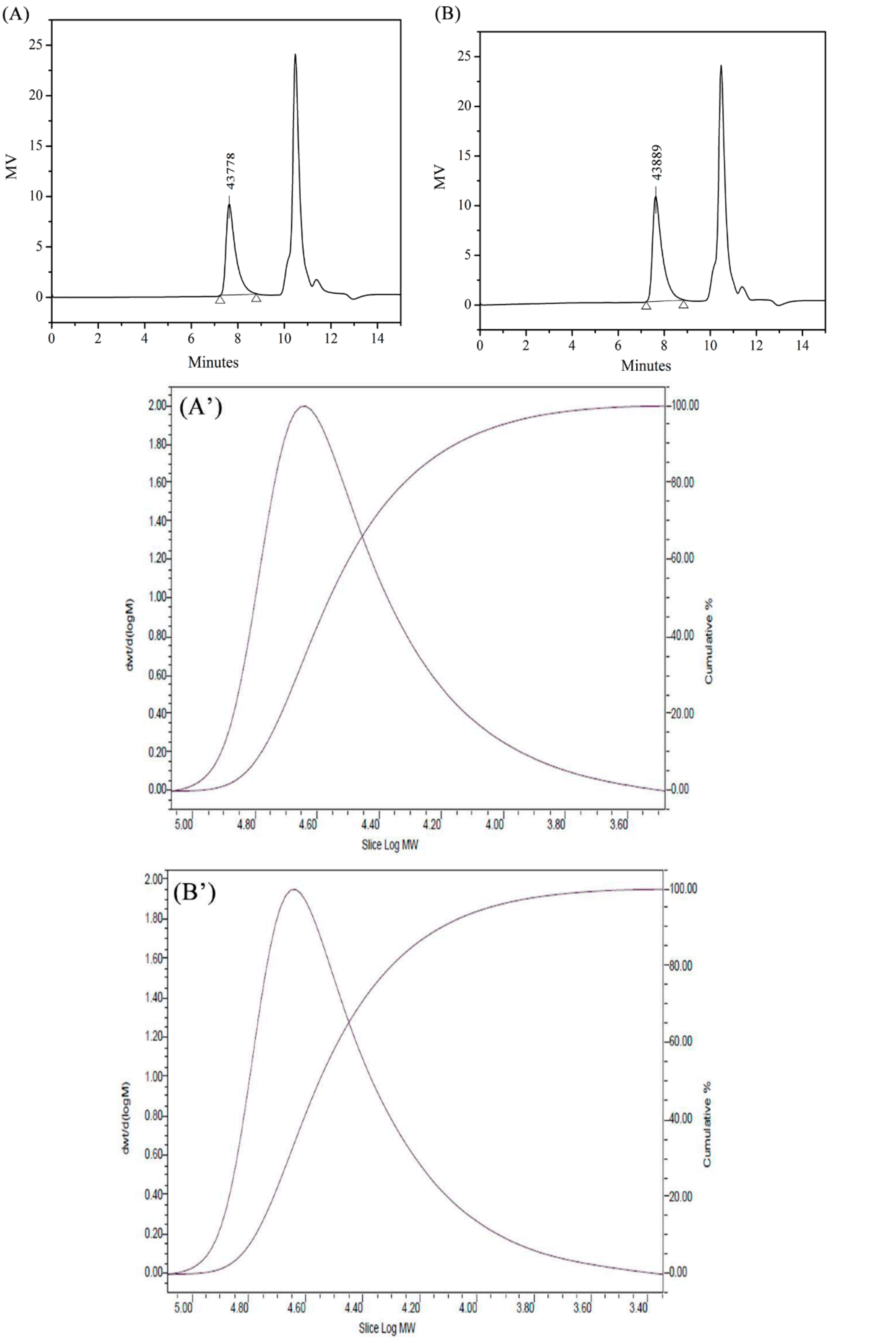

Figure S3. GPC traces of the novel PAI [(**A**,**A′**)] and the pure PAI [(**B**,**B′**)].
